# Physiological and Psychological Response to Acute Mental Stress in Female Patients Affected by Chronic Pulmonary Arterial Hypertension: An Explorative Controlled Pilot Trial

**DOI:** 10.3390/ph16040493

**Published:** 2023-03-27

**Authors:** Alessandra Gorini, Beatrice De Maria, Patrycja Krasinska, Maurizio Bussotti, Francesca Perego, Laura Adelaide Dalla Vecchia

**Affiliations:** 1 Istituti Clinici Scientifici Maugeri IRCCS, 20138 Milan, Italy; 2 Dipartimento di Scienze Cliniche e di Comunità, Università degli Studi di Milano, 20122 Milan, Italy

**Keywords:** pulmonary arterial hypertension, mental stress, heart rate, perceived stress, individualized cardiac rehabilitation

## Abstract

Little is known about physiological and psychological responses to mental stress in stable patients affected by pulmonary arterial hypertension (PAH). The current explorative controlled pilot study was conducted to investigate whether heart rate (HR) and perceived stress would differ during standardized mental stress testing in PAH patients compared to healthy subjects. Correlation analysis between HR, perceived stress, participants’ psychological status and performance on the mental stress task was also performed. The study included 13 female PAH patients (average age: 44.38 ± 10.88 years; average education: 14 ± 3.07 years; mean duration of illness: 9.15 ± 5.37 years) and 13 female controls similar in age (mean age: 47.85 ± 6.36 years) and education (15.92 ± 1.55 years). Participants performed a standardized 9 min mental stress test (computer based, adaptive math task). HR and perceived stress during the task were compared to resting baseline and correlated with psychological state and task performance. Both HR and perceived stress significantly increased during mental stress in a similar way in both groups. A significant correlation was found between HR and perceived stress. Our data show that moderate mental stress has a comparable effect on HR and perceived stress increase in stable PAH patients and control subjects.

## 1. Introduction

Pulmonary arterial hypertension (PAH) is a rare, progressive disorder occurring 3–5 times more frequently in females (mostly between 30 and 60 yrs) than in males and characterized by an increased pulmonary vascular resistance (PVR) leading to right ventricular (RV) failure [1,2].

The female gender seems to represent the most important epidemiological risk factor for the development of PAH: in fact, the analysis of data from the major European [3,4,5] and American international multicenter registries [6,7] confirms a clear prevalence of female patients for all types of PAH, with a female-to-male ratio overall greater than 3:1.

Like other disorders involving right heart failure, such as congenital heart disease, and pulmonary and tricuspid incompetence [8], PAH is generally associated with an increased sympathetic activation, which may adversely affect the overall cardiac autonomic control [9,10,11], leading to a poorer outcome [9,11,12], disease severity [13,14,15] and a higher mortality risk [16,17].

Mental stress is an established trigger to elicit an activation of the sympathetic nervous system and a withdrawal of the parasympathetic one, resulting in a higher heart rate (HR) [18,19] and lower RR interval. In some pathological conditions, including acute and chronic coronary syndrome [20,21,22] and heart failure [23], such normal physiological response may be altered and associated with either exaggerated or blunted cardiovascular reactivity, which is known to significantly impact physical and mental health [24,25,26,27].

In the only available study using mental stress to induce physiological changes in severe PAH, Schachinger and coll. [28] suggested that modulation of sympathetic activity in PAH patients exposed to moderate mental stress may play a role in the control of the pulmonary circulation, causing an elevation of pulmonary arterial pressure and PVR. However, regarding the HR response during the stress test administration, the authors only observed “an obvious HR response in every individual patient, indicating effective autonomic nervous system activation during the task procedure”. Despite the relevance of this study for understanding the effects of stress induction on the pulmonary hemodynamic response, the lack of a matched control group does not allow for making a definitive conclusion about the adequacy of the cardiac response in PAH patients. Moreover, as it is known that HR may also be influenced by the individual stressful situation appraisal [29], it is important to evaluate this aspect in addition to the physiological response.

According to these observations, and to overcome the limitations of the Schachinger study, we conducted a pilot study based on an explorative controlled trial to compare and correlate HR variations, perceived stress, psychological state and performance on a mental stress task in a group of PAH patients and in a matched group of healthy subjects at baseline and during a standardized mental stress condition.

## 2. Materials and Methods

### 2.1. Participants

Thirteen female patients treated for PAH at the Cardiac Rehabilitation Department of the IRCCS Istituti Clinici Scientifici Maugeri in Milan (Italy) participated in the current investigation. The patients’ mean age was 44.38 ± 10.88 yrs; the mean level of education was 14 ± 3.07 yrs; and the mean duration of illness calculated from the diagnosis was 9.15 ± 5.37 yrs.

Patients were selected according to the following criteria:

Inclusion criteria

- Having a diagnosis of PAH;
- Being female (most of the patients of our cohort are females, so the few males were excluded);
- Being ≥18 years-old;
- Having normal or corrected-to-normal hearing and vision;
- Receiving stable PAH therapy from > 6 months;
- Understanding the Italian language and being able to interact with the interviewer.

Exclusion criteria

- Currently taking psychotropic medication or experiencing serious mental illness (e.g., schizophrenia or psychosis);
- Having ascertained cognitive deficits.

Thirteen female healthy subjects of similar age (47.85 ± 6.36 yrs) and education (15.92 ± 1.55 yrs) were enrolled as a control group. The healthy status was defined as absence of any chronic or intercurrent acute disease and of any actual pharmacological treatment. Regular exercise training was an exclusion criterion, as it is known to interfere with both the sympathovagal and psychological profile [30].

The study conformed to the standards set by the Declaration of Helsinki; ethical approval was obtained from the ethical committee of Istituti Clinici Scientifici Maugeri in Pavia (approval number: 2694 CE), and all participants signed a written informed consent.

### 2.2. Patients’ Clinical Characteristics

All the patients involved in the study were affected by Group 1 varieties of PAH (7 idiopathic, 5 associated with congenital heart disease, 1 associated with HIV infection). They were in World Health Organization Functional Class (WHO FC) I (n = 2), II (n = 6) or III (n = 5) and were in a stable clinical condition. PAH diagnosis had been previously confirmed by a right heart catheterization. All patients were under optimal medical therapy, 7 (44%) receiving a dual therapy, and 6 (56%) a triple therapy. Specifically, 5 patients were receiving a combination of a phosphodiesterasis type 5 inhibitor (PHD5i) and an endothelin receptor antagonist (ERA), 1 a combination of riociguat and ERA, 4 a combination of PHD5i–ERA and parenteral prostacyclines, 2 a combination of riociguat–ERA and Selexipag, and 1 a combination of PHD5I–ERA–Selexipag.

Upon enrollment, all participants completed the Short-form Depression Anxiety and Stress Scale (DASS-21) [31], i.e., the short version of the original self-report questionnaire developed and validated by Lovibond et al. [32] to evaluate depression, anxiety and stress symptoms. It is composed of 21 items, with 7 items per subscale, namely, (1) DASS-21 Depression, a specific subscale for assessing depressed mood and absence of positive emotions (e.g., ‘I could not seem to experience any positive feeling at all’); (2) DASS-21 Anxiety, a specific subscale for evaluating anxiety feelings and somatic tension (e.g., ‘I was aware of the dryness of my mouth’); (3) DASS-21 Stress, a specific subscale for evaluating somatic stress, with a focus on difficulty in relaxing and irritability (e.g., ‘I found it hard to wind down’). Participants are asked to score every item on a scale from 0 (‘did not apply to me at all’) to 3 (‘applied to me very much’). In the context of the present study, DASS-21 was administrated to verify if the incidence of depressive, anxious and/or stress symptoms was different in the 2 groups, interfering with perceived and physiological stress.

### 2.3. Experimental Procedures

#### 2.3.1. Baseline Registration and ECG Signal Processing

After the sociodemographic, clinical, and psychological data collection, patients were instrumented with a portable ECG recording device (Faros 360°, MegaElectronics, Bittium, Kuopio, Finland) with a sampling rate fixed at 500 Hz, while sitting on a comfortable chair in a quiet room.

A baseline registration was performed for 5 min before the mental stress induction, with the participants seated in a relaxed position with their eyes closed.

From the recorded ECG signal, the RR interval beat-to-beat time series was derived. The RR interval (RR) was defined as the temporal distance between two consecutive R peaks detected on the ECG. The algorithm applied for the R peak detection was based on a threshold on the first derivative of the ECG signal and allowed for fixing R-wave peaks by means of parabolic interpolation. RR was expressed in ms.

The R-wave peak detections were visually checked to avoid misidentification. In the presence of extrasystoles, the RR was corrected by means of cubic spline interpolation. Attention was paid to never exceed with corrections 5% of the total RR intervals.

The mean of the RRs, the maximum RR and the minimum RR were calculated over the whole period of recording for each experimental condition. To avoid performing analysis over the transitory periods, the first and last 10 s of each study condition were not considered.

#### 2.3.2. Stress Induction

Mental stress was induced using a computer-generated version of the Montreal Imaging Stress Task (MIST) [33] created using the Millisecond Software and validated by multiple studies [34,35,36]. The task consists of a series of arithmetical operations of 5 different levels (levels automatically increase when performance increases and decrease when performance decreases) based on combinations of addition, subtraction, multiplication and division. After a brief training phase, participants were asked to complete 3 blocks of mental operations (MIST1, MIST2 and MIST3), each lasting 3 min. The time limit to complete each operation was set at 5 s. The MIST interface displayed the time limit and the accumulated success rate of the participant as two different progress bars over each operation. The success rate was intentionally displayed to induce pressure on the participant. Additionally, the technician in charge of the experiment entered the room to verbally push the participants on three occasions during the test phase. To complete the MIST, the participants were instructed to remain seated and to use the laptop touchscreen with their dominant hand to indicate the correct result for each operation. The MIST has a total duration of 9 min.

After the baseline and at the end of each of the 3 MIST blocks, multiple Subjective Units of Distress Scale (SUDS) [37] were administered to gather the self-perceived stress level of the participants. Each SUDS consists of a simple numeric rating scale from 0 to 10 that measures the actual level of distress perceived by the individual.

### 2.4. Statistical Analysis

Data were anonymized and analyzed in an aggregated way. Descriptive statistics were used to analyze descriptive variables characterizing the sample. Categorical variables were described as absolute number (percentage), while continuous variables were presented as mean ± standard deviation.

Two-tailed Student t-test, or Mann–Whitney rank test in case of non-normal distribution, was performed to compare the demographic and clinical variables and DASS sub-scores of healthy controls and PAH patients.

Two-way repeated measure analysis of variance (ANOVA, Holm–Sidak test for multiple comparisons) was performed to verify the differences in the ECG-derived, psychological and performance variables between the two groups (i.e., controls vs. PAH patients) within each of the experimental conditions (i.e., baseline vs. MIST).

To test the association between ECG-derived parameters and clinical and psychological variables, Spearman rank correlation coefficient ρ and the probability p of type I error associated with ρ were calculated.

A significance level of 0.05 was set for all the above-mentioned analyses. The analyses were carried out using a commercial software (Sigmaplot, Systat Software, Inc., Chicago, IL, USA, version 11.0).

## 3. Results

Details concerning demographic and clinical characteristics of patients and controls are shown in Table 1.

**Figure 1:**
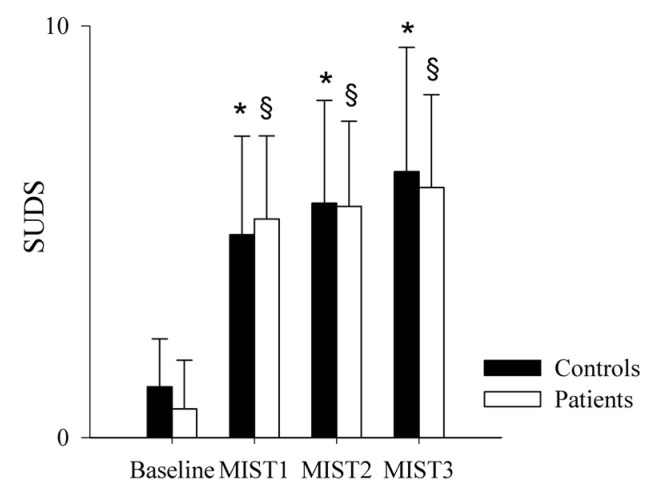
Comparison of the SUDS scale scores in healthy controls and PAH patients during the experimental sessions. The bar graphs show the Subjective Units of Distress Scale (SUDS) scores in healthy controls (black bars) and in PAH patients (white bars) at the end of the resting condition (Baseline) and after each of the three different blocks of the Montreal Imaging Stress Test (MIST1, MIST2 and MIST3). Results are presented as mean ± standard deviation. * indicates *p* < 0.05 vs. Baseline in the control group, and ^§^ indicates *p* < 0.05 vs. Baseline in the PAH patients’ group.

**Figure 2:**
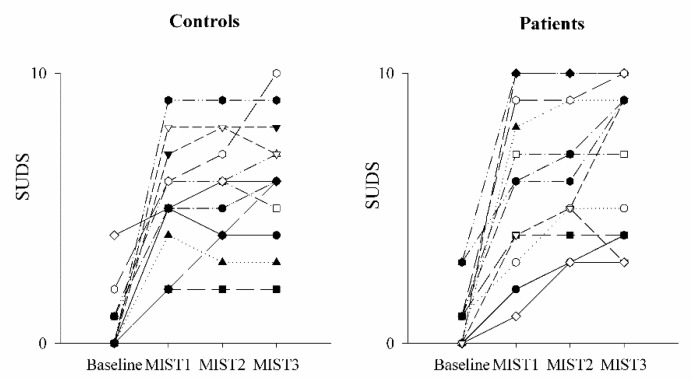
Trend of the SUDS scale scores as a function of the experimental sessions in controls and PAH patients. The scatterplots show the Subjective Units of Distress Scale (SUDS) scores in healthy controls (**left** panel) and in PAH patients (**right** panel) at the end of the resting condition (Baseline) and after each of the three different blocks of the Montreal Imaging Stress Test (MIST1, MIST2 and MIST3).

The comparison of the SUDS scale in healthy controls (black bars) and PAH patients (white bars) as a function of the baseline and experimental conditions (mental stress) is shown in Figure 1 and Figure 2. SUDS scores were higher both in controls and patients during the three math blocks (MIST1, MIST2 and MIST3) compared to the baseline condition.

**Figure 3:**
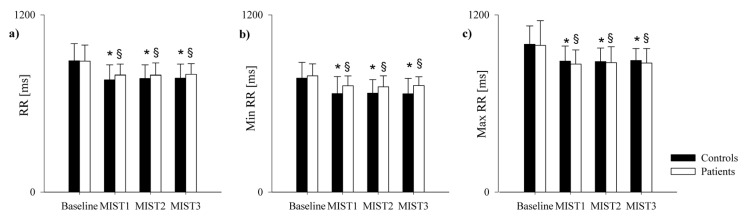
Comparison of the ECG-derived parameters in healthy controls and PAH patients during the experimental sessions. The bar graphs show the mean RR (panel **a**), minimum RR (panel **b**) and maximum RR (panel **c**) recorded in healthy controls (black bars) and in PAH patients (white bars) in resting condition (Baseline) and during the three different blocks of the Montreal Imaging Stress Test (MIST1, MIST2 and MIST3). Results are presented as mean ± standard deviation. * indicates *p* < 0.05 vs. Baseline in the control group, and § indicates *p* < 0.05 vs. Baseline in the PAH patients’ group.

**Figure 4:**
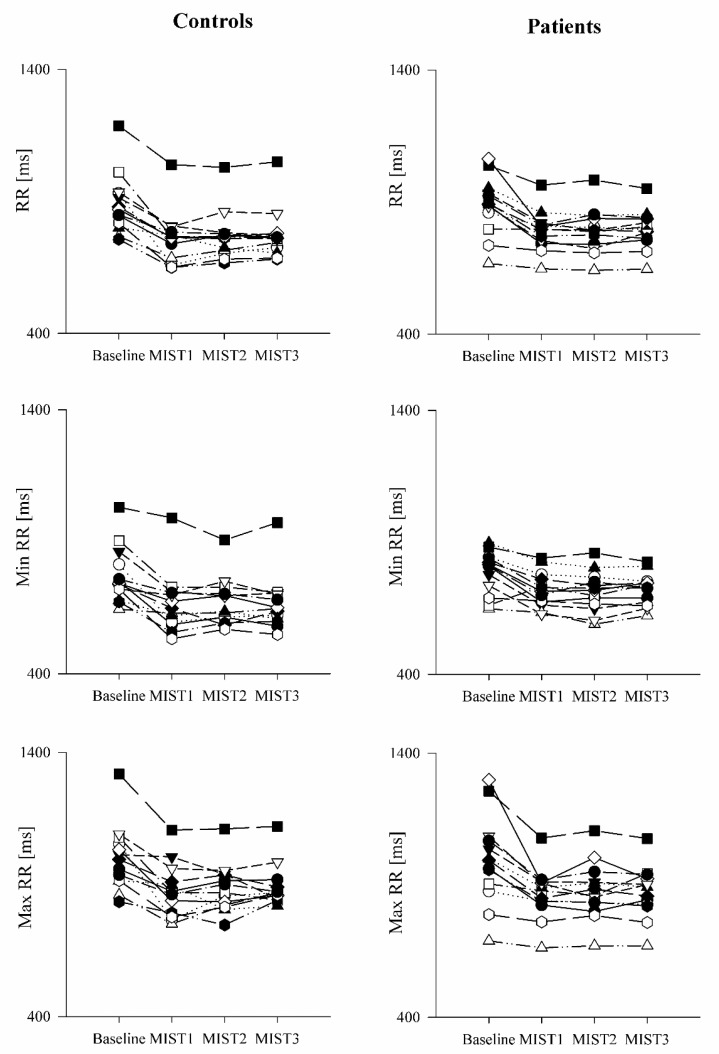
Trend of the ECG-derived parameters as a function of the experimental sessions in healthy controls and PAH patients. The scatter plots show the mean RR (**upper** panels), minimum RR (**middle** panels) and maximum RR (**lower** panels) recorded in healthy controls (**left** panels) and in PAH patients (**right** panels) in resting condition (Baseline) and during the three different blocks of the Montreal Imaging Stress Test (MIST1, MIST2 and MIST3).

The comparison of the ECG-derived parameters in healthy controls and PAH patients is shown in Figure 3 and Figure 4. The mean RR (Figure 3a), minimum RR (Figure 3b) and maximum RR (Figure 3c) were lower during the three math blocks (MIST1, MIST2 and MIST3) compared to the baseline, both in controls and in patients. No significant differences were found between the three blocks and between controls and patients, neither in the SUDS scores or in the ECG-derived parameters.

Due to the lack of significant differences between MIST1, MIST2 and MIST3 both in patients and controls, the following analyses were conducted considering the three blocks as a whole.

**Figure 5:**
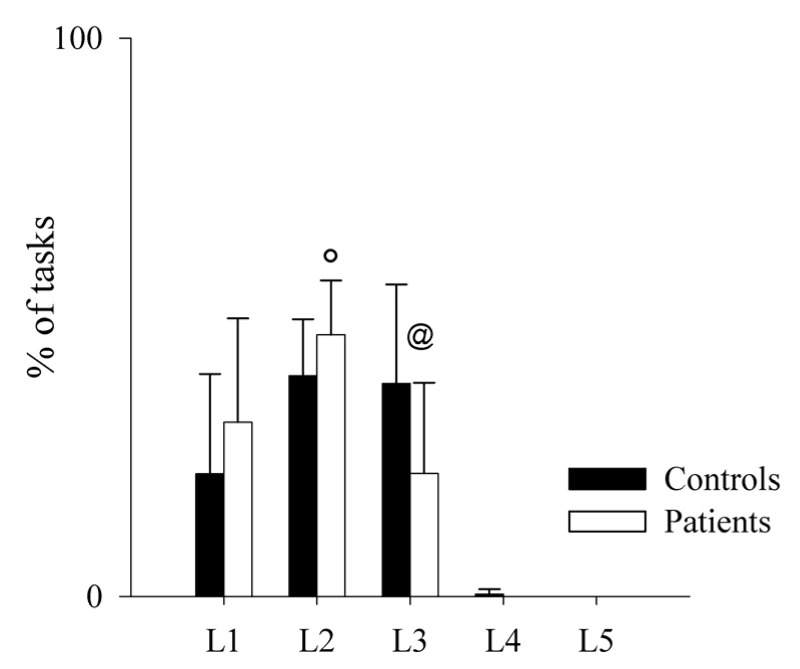
Comparison of the performance on the MIST test in healthy controls and PAH patients. The bar graphs show the percentage of tasks in each level of difficulty during the MIST session in healthy controls (black bars) and PAH patients (white bars). Level 1 (L1) is the easiest, while Level 5 (L5) is the most difficult one. Results are presented as mean ± standard deviation. @ indicates *p* < 0.05 controls vs. patients, and ° indicates *p* < 0.05 Level 3 vs. Level 2 in patients.

**Figure 6:**
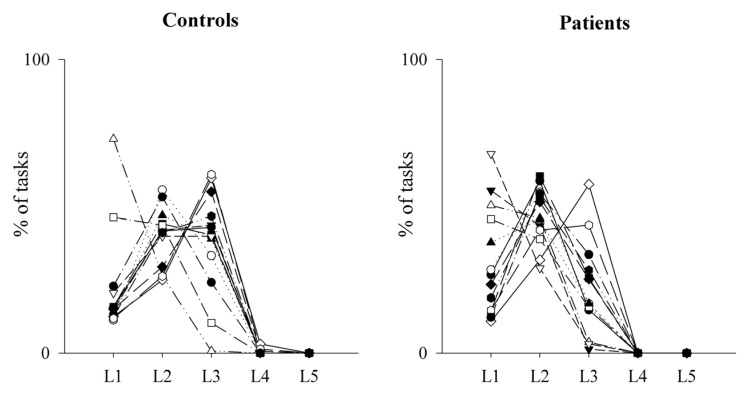
Trend of the performance on the MIST test as a function of the levels reached by healthy controls and PAH patients. The scatterplots show the percentage of tasks at each level of difficulty during the MIST session in healthy controls (**left** panel) and PAH patients (**right** panel). Level 1 (L1) is the easiest, while Level 5 (L5) is the most difficult one.

Regarding the performance on the MIST task (total % of correct answers) and the reaction times (RT) to give the right answers, these did not differ between the two groups (mean % of correct answers: controls = 57.91 ± 4.65; patients: 57.96 ± 1.67; *p* = 0.968); (mean RT: controls = 2566.24 ± 417.54 ms; patients: 2786.51 ± 321.29 ms; *p* = 0.145). Moreover, since the task difficulty automatically increases if performance increases and decreases if performance decreases, we also calculated the total number of operations performed by each group at each level (Figure 5 and Figure 6), finding that controls had a better performance than patients, as shown by the significant higher number of operations solved by the firsts at Level 3.

**Figure 7:**
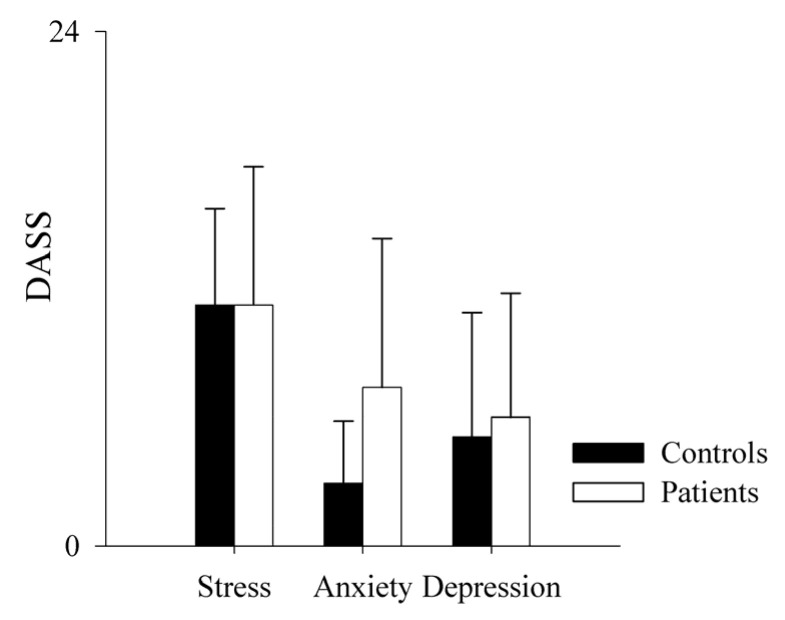
Comparison of the DASS scale sub-scores in healthy controls and PAH patients. The bar graphs show the stress, anxiety and depression subscales of the Depression, Anxiety and Stress Scale (DASS) in healthy controls (black bars) and PAH patients (white bars). Results are presented as mean ± standard deviation.

Regarding the DASS subscales (i.e., stress, anxiety and depression), we found that controls and patients had similar results in the stress (mean controls = 11.23 ± 4.5; mean patients = 11.23 ± 6.46; *p* = 1) and depression (mean controls = 5.08 ± 5.81; mean patients 6 ± 5.77; *p* = 0.528) subscales. Regarding the anxiety subscale, we observed a non-significant tendency to higher values in patients (mean = 7.38 ± 6.95) compared to controls (mean = 2.92 ± 2.9), as shown in Figure 7.

The results of the correlation analysis between performance on MIST and psychological status and ECG-derived parameters at baseline (Table 2) and during MIST (Table 3) did not reveal any correlation.

Finally, we calculated the correlation between physiological data and the SUDS scores, finding that mean RR (*ρ* = −0.527, *p* = 7.01 × 10^−5^), maximum RR (*ρ* = −0.531, *p* = 6.06 × 10^−5^) and minimum RR (*ρ* = −0.354, *p* = 1.03 × 10^−2^) of patients were significantly negatively correlated with the SUDS scores. Similar results were obtained in the control group (mean RR *ρ* = −0.531, *p* = 6 × 10^−6^; maximum RR *ρ* = −0.473, *p* = 4.46 × 10^−4^; and minimum RR *ρ* = −0.471, *p* = 4.64 × 10^−4^) (see Table 2 and Table 3).

## 4. Discussion

Very little is known about the physiological and psychological stress response of PAH patients to mental stress. With the present explorative controlled pilot trial, we observed no significant differences between patients and control subjects in their physical and psychological response to acute mental stress. In both groups, performing mental arithmetic caused a comparable significant increase in the perceived stress (measured with the SUDS) and of heart rate (measured with the ECG signal), indicating that PAH patients show neither the exaggerated nor blunted cardiovascular reactivity that has been commonly observed in patients with cardiovascular diseases.

The mental stress task induced an increased HR (i.e., lower RR) in both controls and patients. This increase was evident from the first block of the MIST and lasted, without significant variations, for the entire duration of the task, indicating a prompt reactivity to the stressful stimuli in both groups. Both the sign and the amount of HR variation were similar in the two groups.

The observed cardiac response is the result of the sympathetic activation commonly observed in healthy subjects after stress induction [38]. A very similar response was also observed by Schachinger and coll. [28] in a sample of 7 PAH patients. Nevertheless, their results needed to be replicated, as the authors did not include in their study a matched sample of healthy subjects for comparison.

Moreover, since HR is known to be also influenced by the individual stressful situation appraisal [29], we analyzed perceived stress and correlated it with physiological data. We did not find any significant difference between patients and healthy subjects in perceived stress induced by the mental task, and found a significant correlation in both groups between physiological and perceived stress, indicating a coherence between physical response and self-reported perception.

The observed lack of differences between patients and controls suggests that the cardiac response mechanism to acute mental stress is not exaggerated in the presence of PAH, differently to what happens in other cardiac diseases [24]. However, the findings of the present study differ from the literature, showing a similar response in patients and controls. This discrepancy could be explained by the fact that the enrolled patients were in optimal therapy and hemodynamically stable and/or by the fact that the mental stress stimulus was not sufficiently intense to elicit an exaggerated or blunted response in patients.

Regarding the DASS-21 data, we only observed a trend toward higher anxiety in the patient sample compared to healthy subjects, which is only partially in line with previous studies about psychological burden affecting PAH patients [39]. This may be mainly due to the stable and well-compensated clinical status of the patients enrolled in the present study.

The only significant differences we found between the two groups regarded the participants’ performance on the MIST. As described above, this task is built to adapt to the responder’s ability to correctly solve the presented math operations (i.e., when the answer is correct, the next item becomes more difficult, and vice versa). Because of this test structure, good responders are presented more difficult stimuli, while bad performers are presented the easiest ones. This may explain why no significant differences were found in the total percentage of correct answers, as well as in the correct answer reaction time between the two groups. Nevertheless, analyzing the number of math operations presented in each level by the two groups, it was evident that patients had a worse performance compared to healthy subjects. Since this difference cannot be attributable to the age or educational level, which did not significantly differ between the two groups, we can hypothesize that it might depend on some mild cognitive impairment as described in previous neuropsychological studies [40], potentially related to a reduced oxygen delivery and cerebral tissue oxygenation, neuroinflammation, and/or dysregulation of neurodegenerative factors [41] often characterizing PAH patients and also reported by the patients themselves.

To our knowledge, this is the first pilot study comparing the physiological and psychological stress response elicited by acute mental stress in PAH patients and control subjects. Results should be confirmed by a larger sample, possibly including males, as it is known that the stress reaction may be different in men and women [42]. A higher sample size would help understand the impact of disease severity on the results by stratifying patients by clinical variables. Furthermore, unstable patients should be enrolled in order to identify the possible target population that could benefit from interventions, such as relaxation sessions, aimed at reducing adrenergic hyperactivity. As previously described, sessions of slow breathing and muscular relaxation were able to improve quality of life and ameliorate ventilatory efficiency [43].

In conclusion, this study showed that PAH patients have a normal or pseudo-normal response to acute stress. This is in contrast with data obtained in other cardiac conditions, reinforcing the fact that the PAH patients have peculiar aspects requiring specific therapeutic approaches, including training and rehabilitation programs. In fact, even in PAH patients with a high impairment of functional capacity, specific training has shown some benefits [1].

## Figures and Tables

**Table 1:** Demographic and clinical characteristics of the enrolled samples.

	Controls (n = 13)	PAH Patients (n = 13)
Age, yrs	47.8 ± 6.365	44.38 ± 10.88
Gender, females	13 (100)	13 (100)
BMI, kg·m^2^	24.35 ± 5.39	25.66 ± 7.78
PAH Group 1 etiology		
Idiopathic		7 (54)
Associated with congenital cardiopathy		5 (38)
HIV-related		1 (8)
WHO Functional Class		
I		2 (15)
II		6 (46)
IIIa		3 (23)
IIIb		2 (15)
mPAP, mmHg		50.08 ± 18.01
PVR, WU		8.05 ± 5.25
CI, L/min/m^2^		2.81 ± 0.79
Pharmacological therapy		
5PHDi		10 (77)
Riociguat		3 (23)
ERA		13 (100)
Prostacyclin		4 (31)
Selexipag		3 (23)

PAH, pulmonary arterial hypertension; BMI, body mass index; WHO, World Health Organization; mPAP, mean pulmonary artery pressure; PVR, arteriolar pulmonary resistance; WU, Wood Unit; CI, cardiac index; 5PHDi, phosphodiesterasis type 5 inhibitor; ERA, endothelin receptor antagonist. Continuous data are presented as mean ± standard deviation, while categorical ones as number (percentage).

**Table 2:** Results of the correlation analysis between the ECG-derived parameters and performance and psychological variables in patients and controls at baseline.

	Mean RR [ms]		Minimum RR [ms]		Maximum RR [ms]	
	*ρ*	*p*	*ρ*	*p*	*ρ*	*p*
Controls						
Correct answers, %	0.071	0.806	0.071	0.806	0.124	0.669
Reaction time, ms	−0.636	0.214	−0.181	0.541	−0.407	0.160
Tasks in Level 1, %	0.220	0.458	0.093	0.751	0.173	0.553
Tasks in Level 2, %	0.313	0.286	0.505	0.074	0.234	0.424
Tasks in Level 3, %	−0.143	0.629	−0.093	0.751	−0.093	0.751
DASS stress	0.192	0.516	0.022	0.935	0.131	0.656
DASS anxiety	−0.208	0.481	−0.316	0.278	−0.177	0.553
DASS depression	0.008	0.964	0.180	0.541	−0.036	0.892
Patients						
Correct answers, %	0.448	0.116	0.462	0.107	0.160	0.591
Reaction time, ms	−0.082	0.778	−0.181	0.541	0.001	0.993
Tasks in Level 1, %	−0.445	0.121	−0.401	0.166	−0.291	0.323
Tasks in Level 2, %	0.132	0.656	0.505	0.074	−0.099	0.737
Tasks in Level 3, %	0.341	0.244	0.368	0.206	0.176	0.553
DASS stress	0.205	0.493	0.118	0.696	0.283	0.332
DASS anxiety	−0.318	0.278	−0.122	0.682	−0.379	0.192
DASS depression	−0.181	0.541	0.142	0.629	−0.150	0.616

RR, RR interval; DASS, short-form Depression Anxiety and Stress Scale; *ρ*, Pearson product moment correlation coefficient; *p*, probability of type I error associated with *ρ*.

**Table 3:** Results of the correlation analysis between the ECG-derived parameters and performance and psychological variables in patients and controls during MIST.

	Mean RR [ms]		Minimum RR [ms]		Maximum RR [ms]	
	*ρ*	*p*	*ρ*	*p*	*ρ*	*p*
Controls						
Correct answers, %	0.159	0.591	−0.132	0.656	0.418	0.148
Reaction time, ms	−0.115	0.696	−0.126	0.669	−0.154	0.603
Tasks in Level 1, %	0.505	0.074	0.654	0.064	0.374	0.199
Tasks in Level 2, %	0.154	0.603	0.214	0.469	0.187	0.528
Tasks in Level 3, %	−0.104	0.723	−0.396	0.173	0.049	0.863
DASS stress	0.381	0.132	0.281	0.132	0.310	0.295
DASS anxiety	−0.048	0.863	0.202	0.493	−0.351	0.229
DASS depression	−0.107	0.723	0.042	0.0878	−0.351	0.229
Patients						
Correct answers, %	0.300	0.304	0.410	0.154	0.135	0.643
Reaction time, ms	0.001	0.993	−0.170	0.565	0.011	0.964
Tasks in Level 1, %	−0.346	0.236	−0.500	0.078	−0.308	0.295
Tasks in Level 2, %	0.198	0.504	0.495	0.081	−0.049	0.863
Tasks in Level 3, %	0.286	0.332	0.489	0.081	0.225	0.447
DASS stress	0.140	0.629	0.236	0.424	0.208	0.481
DASS anxiety	0.019	0.935	0.141	0.629	−0.058	0.835
DASS depression	0.108	0.709	0.345	0.236	0.011	0.964

RR, RR interval; DASS, short-form Depression Anxiety and Stress Scale; *ρ*, Pearson product moment correlation coefficient; *p*, probability of type I error associated with *ρ*.

## Data Availability

Data contained in this study are available on reasonable request to the corresponding author.
